# Non-invasive mapping of cortical categorization function by repetitive navigated transcranial magnetic stimulation

**DOI:** 10.1038/s41598-021-04071-4

**Published:** 2021-12-29

**Authors:** Stefanie Maurer, Vicki Marie Butenschoen, Bernhard Meyer, Sandro M. Krieg

**Affiliations:** 1 Department of Neurosurgery, Klinikum rechts der Isar, Technische Universität München, Ismaningerstr. 22, 81675 Munich, Germany; 2 TUM-Neuroimaging Center, Klinikum rechts der Isar, School of Medicine, Technische Universität München, Ismaningerstr. 22, 81675 Munich, Germany

**Keywords:** Cognitive control, Neural circuits

## Abstract

Over the past years navigated repetitive transcranial magnetic stimulation (nrTMS) had become increasingly important for the preoperative examination and mapping of eloquent brain areas. Among other applications it was demonstrated that the detection of neuropsychological function, such as arithmetic processing or face recognition, is feasible with nrTMS. In order to investigate the mapping of further brain functions, this study aims to investigate the cortical mapping of categorization function via nrTMS. 20 healthy volunteers purely right-handed, with German as mother tongue underwent nrTMS mapping using 5 Hz/10 pulses. 52 cortical spots spread over each hemisphere were stimulated. The task consisted of 80 pictures of living and non-living images, which the volunteers were instructed to categorize while the simulation pulses were applied. The highest error rates for all errors of all subjects were observed in the left hemisphere’s posterior middle frontal gyrus (pMFG) with an error rate of 60%, as well as in the right pMFG and posterior supra marginal gyrus (pSMG) (45%). In total the task processing of non-living objects elicited more errors in total, than the recognition of living objects. nrTMS is able to detect cortical categorization function. Moreover, the observed bihemispheric representation, as well as the higher error incidence for the recognition of non-living objects is well in accordance with current literature. Clinical applicability for preoperative mapping in brain tumor patients but also in general neuroscience has to be evaluated as the next step.

## Introduction

The advanced and non-invasive method of navigated repetitive transcranial magnetic stimulation (nrTMS) gained broad interest in the neuroscientific community due to its successful examination of various cortical brain functions. Particularly the examination of eloquent motor and language-related areas was of great interest^1^. In this context, besides motor and language-related function, studies about various neuropsychological functions and their cortical distribution were published using nrTMS recently. Cortical Calculation function, face processing or prosopagnosia and the development of neglect-like deficits were already successfully examined and located in healthy volunteers^2–5^. Moreover, Ille et al. were able to examine the cortical distribution of calculation function pre- and postoperatively in brain tumor patients^6^.

The hypothesis of the current pilot study is that nrTMS detects cortical areas involved in processing of the neuropsychological function of categorization in healthy subjects.

## Results

### Error rate relative to all stimulations

#### Error distribution for all error types

**Figure 1:**
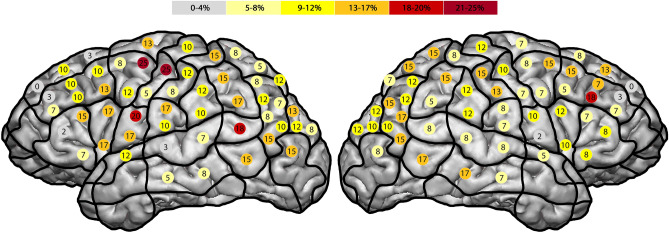
Total amount of generated errors for all errors of all stimulations. The highest ER of 18%^7^ was found in the right mMFG. In comparison, the left hemisphere generated the highest ER of 25% (dark red) in the pMFG and the mPrG.

**Figure 2:**
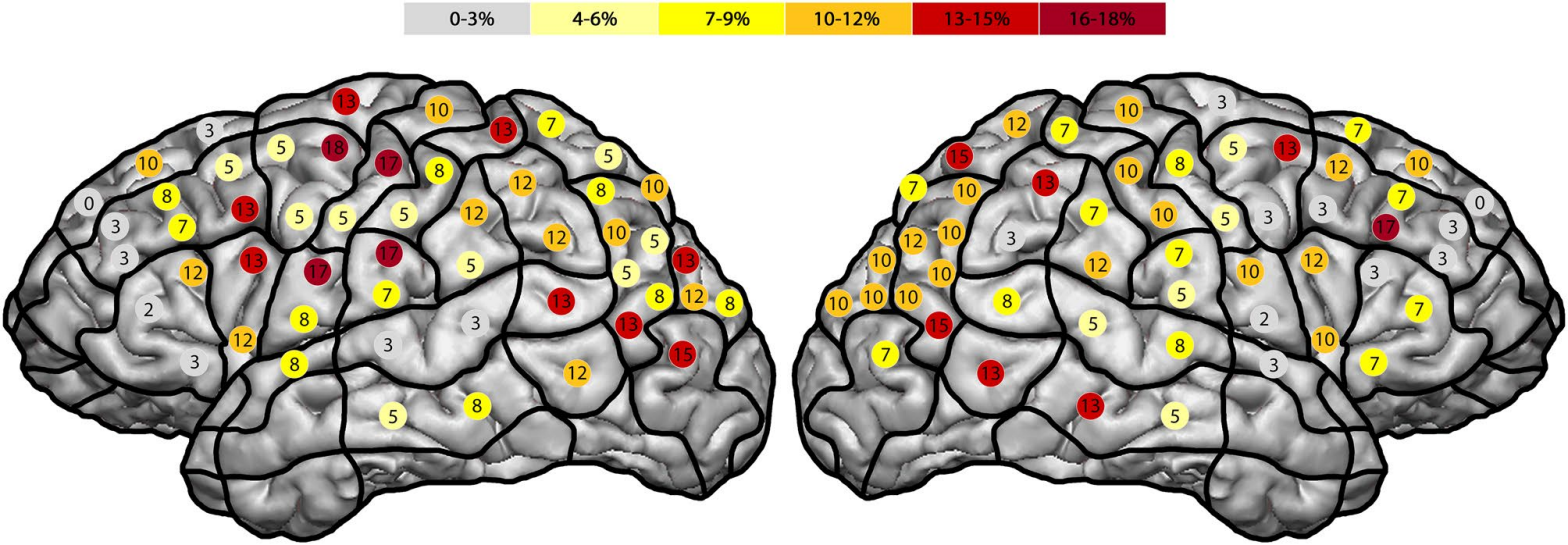
All observed hesitation errors concerning all errors of all stimulations. The highest ER of the right hemisphere was 17% in the mMFG, as well as 18% in the left pMFG.

Concerning the right hemisphere, we observed the highest error rate (ER) of 18% in the middle middle frontal gyrus (mMFG) (SP 8) (Fig. 1). In comparison, the left hemisphere generated the highest ER of 25% in the posterior middle frontal gyrus (pMFG) (SP 18) and the middle pre-central gyrus (mPrG) (SP 20) (Table 1). Overall, the right hemisphere generated an ER of 10%, compared to 11% regarding the left hemisphere. Comparing all errors for all generated stimulations in total in the left vs. the right hemisphere we could not show any statistical significance (p-value: 0.192), however Fig. 2 illustrates a trend regarding a higher ER in the left hemisphere.

**Table 1:** Different errors per stimulation points.

Stimulation spot	No response		Hesitation		Wrong living		Wrong non-living		All errors	
	Errors	Ratio	Errors	Ratio	Errors	Ratio	Errors	Ratio	Errors	Ratio
**(a) Left hemisphere**										
1	0	0.00	0	0.00	0	0.00	0	0.00	0	0.00
2	0	0.00	2	0.03	0	0.00	0	0.00	2	0.03
3	0	0.00	2	0.03	2	0.03	0	0.00	4	0.07
4	0	0.00	1	0.02	0	0.00	0	0.00	1	0.02
5	0	0.00	2	0.03	1	0.02	1	0.02	4	0.07
6	0	0.00	6	0.10	0	0.00	0	0.00	6	0.10
7	1	0.02	5	0.08	0	0.00	0	0.00	6	0.10
8	1	0.02	4	0.07	0	0.00	1	0.02	6	0.10
9	1	0.02	7	0.12	0	0.00	1	0.02	9	0.15
10	2	0.03	7	0.12	1	0.02	0	0.00	10	0.17
11	0	0.00	2	0.03	0	0.00	0	0.00	2	0.03
12	2	0.03	3	0.05	0	0.00	1	0.02	6	0.10
13	0	0.00	8	0.13	0	0.00	0	0.00	8	0.13
14	0	0.00	3	0.13	0	0.00	2	0.03	10	0.17
15	0	0.00	3	0.13	0	0.00	0	0.00	8	0.13
16	1	0.02	3	0.05	0	0.00	1	0.02	5	0.08
17	3	0.05	3	0.05	0	0.00	1	0.02	7	0.12
18	2	0.03	11	0.18	1	0.02	1	0.02	15	0.25
19	0	0.00	6	0.10	0	0.00	0	0.00	6	0.10
20	4	0.07	10	0.17	0	0.00	1	0.02	15	0.25
21	0	0.00	3	0.05	0	0.00	0	0.00	3	0.05
22	2	0.03	10	0.17	0	0.00	0	0.00	12	0.20
23	5	0.08	5	0.08	0	0.00	0	0.00	10	0.17
24	0	0.00	5	0.08	1	0.02	1	0.02	7	0.12
25	1	0.02	8	0.13	0	0.00	0	0.00	9	0.15
26	1	0.02	5	0.08	0	0.00	1	0.02	7	0.12
27	0	0.00	3	0.05	2	0.03	0	0.00	5	0.08
28	0	0.00	10	0.17	0	0.00	0	0.00	10	0.17
29	1	0.02	4	0.07	0	0.00	1	0.02	6	0.10
30	0	0.00	2	0.03	0	0.00	0	0.00	2	0.03
31	0	0.00	3	0.05	0	0.00	0	0.00	3	0.05
32	0	0.00	7	0.12	0	0.00	0	0.00	7	0.12
33	2	0.03	3	0.05	0	0.00	1	0.02	6	0.10
34	1	0.02	2	0.03	0	0.00	1	0.02	4	0.07
35	0	0.00	5	0.08	0	0.00	0	0.00	5	0.08
36	1	0.02	7	0.12	0	0.00	1	0.02	9	0.15
37	1	0.02	7	0.12	0	0.00	2	0.03	10	0.17
38	2	0.03	8	0.13	1	0.02	0	0.00	11	0.18
39	1	0.02	7	0.12	1	0.02	0	0.00	9	0.15
40	0	0.00	5	0.08	0	0.00	0	0.00	5	0.08
41	0	0.00	6	0.10	1	0.02	0	0.00	7	0.12
42	2	0.03	3	0.05	0	0.00	0	0.00	5	0.08
43	0	0.00	8	0.13	1	0.02	0	0.02	9	0.15
44	0	0.00	4	0.07	0	0.00	1	0.00	5	0.08
45	1	0.02	3	0.05	0	0.00	0	0.00	4	0.07
46	0	0.00	5	0.08	1	0.02	0	0.00	6	0.10
47	0	0.00	9	0.15	0	0.00	0	0.00	9	0.15
48	0	0.00	3	0.05	0	0.00	0	0.00	3	0.05
49	0	0.00	8	0.13	0	0.00	0	0.00	8	0.13
50	1	0.00	7	0.12	0	0.00	0	0.00	7	0.12
51	0	0.02	6	0.10	0	0.00	0	0.00	7	0.12
52	0	0.00	5	0.08	0	0.00	0	0.00	5	0.08
Median	0.0	0.00	5.0	0.01	0.0	0.00	0.0	0.00	6.0	0.10
Min	0.0	0.00	0.0	0.0	0.0	0.00	0.0	0.00	0.0	0.00
Max	5.0	0.08	11.0	0.18	2.0	0.02	2.0	0.03	15.0	0.25
SD	0.84	0.01	2.22	0.00	0.40	0.00	0.50	0.00	2.47	0.05
**(b) Right hemisphere**										
1	0	0.00	0	0.00	0	0.00	0	0.00	0	0.0
2	0	0.00	2	0.03	0	0.00	0	0.00	2	0.03
3	0	0.00	2	0.03	1	0.02	1	0.02	4	0.07
4	0	0.00	4	0.07	1	0.02	0	0.00	5	0.08
5	0	0.00	4	0.07	0	0.00	1	0.02	5	0.08
6	1	0.02	6	0.10	0	0.00	1	0.02	8	0.13
7	0	0.00	4	0.07	0	0.00	0	0.00	4	0.07
8	1	0.02	10	0.17	0	0.00	0	0.00	11	0.18
9	2	0.03	2	0.03	0	0.00	0	0.00	4	0.07
10	0	0.00	6	0.10	0	0.00	0	0.00	6	0.10
11	1	0.02	4	0.07	0	0.00	0	0.00	5	0.08
12	2	0.03	7	0.12	0	0.00	0	0.00	9	0.15
13	0	0.00	2	0.03	0	0.00	1	0.02	3	0.05
14	0	0.00	7	0.12	0	0.00	0	0.00	7	0.12
15	1	0.02	2	0.03	1	0.02	0	0.00	4	0.07
16	0	0.00	8	0.13	1	0.02	0	0.00	9	0.15
17	1	0.02	2	0.03	0	0.00	0	0.02	4	0.07
18	1	0.02	3	0.05	1	0.02	1	0.00	5	0.08
19	1	0.02	6	0.10	0	0.00	0	0.00	7	0.12
20	1	0.02	5	0.08	0	0.00	0	0.00	6	0.10
21	0	0.00	3	0.05	0	0.00	0	0.02	4	0.07
22	0	0.00	6	0.10	0	0.00	1	0.00	6	0.10
23	0	0.00	1	0.02	0	0.00	0	0.00	1	0.02
24	0	0.00	2	0.03	0	0.00	1	0.02	3	0.05
25	0	0.00	4	0.07	1	0.02	0	0.00	5	0.08
26	0	0.00	6	0.10	1	0.02	2	0.03	9	0.15
27	0	0.00	6	0.10	1	0.02	1	0.02	8	0.13
28	1	0.02	4	0.07	0	0.00	0	0.00	5	0.08
29	0	0.00	3	0.05	0	0.00	1	0.02	4	0.07
30	0	0.00	5	0.0	0	0.00	2	0.00	5	0.08
31	0	0.00	3	0.05	0	0.00	1	0.02	4	0.07
32	1	0.02	4	0.07	1	0.02	1	0.02	7	0.12
33	0	0.00	7	0.12	0	0.00	0	0.00	7	0.12
34	2	0.03	3	0.05	0	0.00	0	0.00	5	0.08
35	2	0.03	8	0.13	0	0.00	0	0.00	10	0.17
36	0	0.00	8	0.13	1	0.02	0	0.00	9	0.15
37	1	0.02	2	0.03	0	0.00	0	0.00	3	0.05
38	0	0.00	5	0.08	0	0.00	0	0.00	5	0.08
39	1	0.02	8	0.13	1	0.02	0	0.00	10	0.17
40	1	0.02	6	0.10	0	0.00	0	0.00	7	0.12
41	1	0.02	6	0.10	0	0.00	0	0.00	7	0.12
42	2	0.03	6	0.10	2	0.03	0	0.00	10	0.17
43	0	0.00	9	0.15	0	0.00	0	0.00	9	0.15
44	2	0.03	7	0.12	0	0.00	0	0.00	9	0.15
45	0	0.00	7	0.12	1	0.02	1	0.02	9	0.15
46	0	0.00	6	0.10	0	0.00	0	0.00	6	0.10
47	0	0.00	4	0.07	1	0.02	0	0.00	5	0.08
48	0	0.00	9	0.15	0	0.00	0	0.00	9	0.15
49	1	0.02	6	0.10	0	0.00	0	0.00	7	0.12
50	0	0.00	6	0.10	0	0.00	0	0.00	6	0.10
51	0	0.00	4	0.07	0	0.00	0	0.00	4	0.07
52	1	0.02	4	0.10	0	0.00	0	0.00	7	0.12
Median	0.0	0.400	5.0	0.09	0.0	0.00	0.0	0.00	6.0	0.10
Min	0.0	0.00	0.0	0.00	0.0	0.00	0.0	0.00	0.0	0.00
Max	2.0	0.03	10.0	0.17	2.0	0.03	1.0	0.03	11.0	0.18
SD	0.70	0.01	2.27	0.04	0.50	0.01	0.50	0.01	2.45	0.04

Summary of different errors types induced by nrTMS stimulation trains per stimulation spot. (a) Errors and error ratio found in the whole left hemisphere. (b) Errors and error ratio observed in the whole right hemisphere.

#### No categorization possible (no response during the stimulation)

The highest ER was 3% in the triangular inferior frontal gyrus (trIFG) (SP 9), the mMFG (SP12), the middle superior temporal gyrus (mSTG) (SP 34), the middle middle temporal gyrus (mMTG) (SP 35), the superior parietal lobe^8^ (SP 44) and the angular gyrus^9^ (SP 46) (Table 1). In the left hemisphere the highest ER was 8% in the vPrG (SP 23). Both hemispheres generated a total ER of 1%. Concerning the statistical analysis, the right hemisphere generated more errors in total than the left hemisphere without showing any statistical significance (p-value: 0.584). We evaluated all generated mistakes, including hesitation errors or no response during the stimulation, although they are more related to speech function in general.

#### Hesitation errors

The highest ER of the right hemisphere concerning this rather speech impairment related error category was 17% in the mMFG (SP 8), as well as 18% in the left pMFG (SP 18) (Fig. 2). In total, the right hemisphere generated 8% and the left hemisphere 9% ER concerning all errors of all stimulations. Statistically there was no significant difference between the two hemispheres (p-value: 0.556).

#### Wrongly categorized living objects

The highest ER in the right hemisphere was located in the aNG (SP 42) (3%). For the left hemispheres, we identified an ER of 3% in the mMFG (SP 3) and the middle post-central gyrus (mPoG) (SP 27). Statistically, using the Mann–Whitney test, there was no significant difference comparing both hemispheres (p-value: 0.626). Nonetheless, there is a trend for more generated errors concerning wrongly categorized living objects in the left hemisphere.

#### Wrongly categorized non-living objects

In terms of the right hemisphere, we achieved an ER of 3% in the mPoG (SP 26) as well as an ER of 3% in the left opercular inferior frontal gyrus (opIFG) (SP 14) and the posterior supramarginal gyrus (pSMG) (SP 37). Statistically, comparing the two hemispheres in terms of categorization of non-living objects using the Mann–Whitney test, the p-value was 0.422.

### Subjects with errors per stimulated subjects

#### Error distribution for all error types

**Figure 3:**
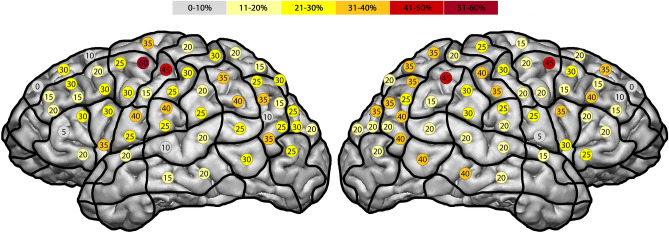
Comparing the error distribution for all error types of all subjects, the highest ER in the right hemisphere was 45% in the pMFG and the pSMG. Additionally, we generated a maximum ER in the left hemisphere of 60% in the pMFG.

The ER in the right hemisphere was 45% in the pMFG (SP 16) and the pSMG (SP 36) (Fig. 3). Additionally, we generated a maximum ER in the left pMFG (SP 16) of 60%. The right hemisphere generated a total ER of 26%, as well as 24% in the left hemisphere. Comparing the two hemispheres using the Mann–Whitney test, the p-value was 0.84.

#### No categorization possible (no response during the stimulation)

The highest ER of 10% was seen in several spots in the right trIFG (SP 9), mMFG (SP 12), mSTG (SP34), mMTG (SP 35), anG (SP 42) and SPL (SP 44). The highest ER of 15% in the left hemisphere was generated in the mPrG. Comparing both hemispheres, the p-value was 0.94.

#### Hesitation errors

**Figure 4:**
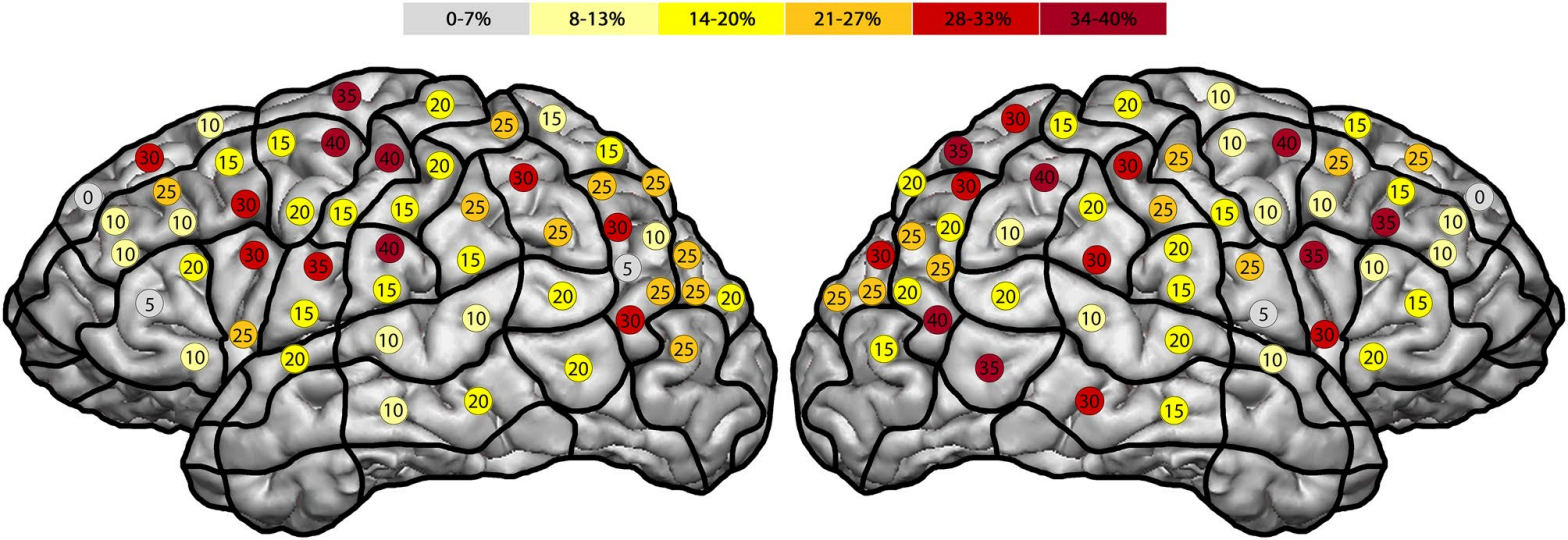
This figure shows the templates with all generated hesitation errors per subjects. The highest ER of this type was observed in the right pMFG^10^ and the anG. In terms of the left hemisphere we observed the highest ER of 40% as well in the pMFG, mPrG and the vPoG.

The highest ER of this type was observed in the right pMFG (SP 16), posterior supramarginal gyrus (pSMG) (SP 36) and the anG (SP 43) (Fig. 4). In the left hemisphere we observed the highest ER of 40% as well in the pMFG (SP 18), mPrG (SP 20) and the ventral post-central gyrus (vPoG) (SP 28). Again, comparing both hemispheres, the right generated ER of 21%, and the left of 20%. The p-value comparing both hemispheres was 0.70.

#### Wrongly categorized living objects

We were able to observe the highest ER of 10% in the right anG (SP 42). In respect of the left hemisphere we found ER of 10% as well in the mMFG (SP 3) and in the mPoG (SP 27). Comparing both hemispheres with the Mann–Whitney-test the p-value was 0.634.

#### Wrongly categorized non-living objects

In terms of this error category the highest ER (10%) was generated in the right mPoG (SP 26) and in the left hemisphere’s pSMG (SP 37). Statistically, the p-value was 0.47.

## Discussion

Besides being biased by additional nrTMS-induced impairment of language function, this study showed the feasibility of disrupting cortical categorization function by nrTMS. Yet, this study is unable to show, at which step of processing, nrTMS is affecting function. In this case, it is difficult to especially distinguish, whether the categorization-function is disrupted via nrTMS during the processing of an incoming stimulus (picture of a living or non-living object), the comparison of the stimulus with an internal category boundary (or a well-known prototype), the decision in terms of categorization itself or the articulation of the result with the following evaluation of the decision^11^. Mendoza et al. pointed out that categorization function depends on the allocation and assignment of various stimuli to specified groups. To answer this complex issue the study group recorded the activity of pre-SMA (supplementary motor area) neurons of monkeys while performing a categorization task. They concluded that the pre-SMA contains important neuronal information to categorize intervals and to evaluate the decision/outcome.

After this initial pilot study, future study designs need to pay close attention on these questions.

Concerning the feasibility of locating cortical regions in association with categorization function, Gross et al. suggested that categorization in terms of object category selectivity was located in the inferior temporal cortex of monkeys in 1972^12^. They continued to discuss whether there are specific locations in the brain, or specific cells, which are responsible for the storage and analysis of all the visual information. Regarding the observed errors in which the subject was not able to categorize a visually displayed picture, our study identified their location in the right hemisphere’s superior- (sMTG) and middle middle temporal gyrus (mMTG) (Fig. 4). Despite the association with speech related errors, this specific error category had more error positive cortical spots located in non-speech dominant areas of the right hemisphere.

Current literature debates several tractography-based language pathways including different parts of the cortex and deeper brain areas. Tuncer et al. discussed five different language pathways including the Fronto-Occipital Fasciculus, the Inferior Longitudinal Fasciculus, the Uncinate Fasciculus, the Frontal Aslant Tract and the Arcuate Fasciculus^13^. In particular, just the Frontal Aslant Tract, relevant for the processing of articulation, involves the middle frontal sulcus and therefore parts of the posterior middle frontal gyrus (highest error rates in the current study). The other language associated pathways involve diverse other parts of the brain like the temporo-parietal segment, the superior frontal sulcus, the circular sulcus of insula, the limen insulae and the parieto-occipital segment. Most important seems the Arcuate Fasciculus located in the inferior frontal lobe and the caudal temporal cortex. In terms of these areas, the highest errors rates in our study occurred in other localizations.

In conclusion, the subject possibly was not able to categorize the picture in these cases due to a categorization-related impairment and less to a speech-related impairment. Via nrTMS, it is generally not possible to reach and stimulate parts of the inferior temporal gyrus^14,15^. Nevertheless, our observed localizations in the temporal lobe confirm the importance of this brain area in terms of categorization-function.

In 1983, Mishkin et al. examined the brains of monkeys and discovered two different multi-synaptic corticocortical pathways concerning the visual and object categorization^16^. The first pathway, located ventrally with connection to the striate and the inferior temporal areas, enables the visual identification of objects. Again, the striate cortex, located in the posterior occipital lobe, and the inferior part of the temporal lobe are areas which are not possible to map with nrTMS due to their accessibility. The second pathway, according to Mishkin et al., runs dorsally with connection to striate and inferior parietal areas. It enables the visual location of objects. Concerning the inferior part of the parietal lobe, we were able to generate an amount of hesitation errors of all subjects in the inferior part of the anG (40% in the right hemisphere and 30% in the left hemisphere) (Fig. 4). Again, hesitation errors are speech-related, but in this special case, the greatest amount of hesitation errors was observed in the parietal lobe of the right hemisphere. This is another indicator for the importance of this generally speech-related errors during the mapping of neuropsychological functions as well, especially when they are not located in typically speech-related cortical areas. In terms of all errors for all subjects we even were able to disturb categorization function in 40% of all subjects in the right inferior part of the anG, as well as in 35% of the left inferior part of the anG (Fig. 3). In this case, our data confirms the additional importance of the inferior part of the parietal lobe in relation to categorization-function and therefore, the possibility of locating cortical categorization function via nrTMS.

Bracci et al. demonstrated that fMRI studies identify the importance of several cortical areas in the response to objects of particular categories^17^. Particular importance was found in the extrastriate body area^6^, in the posterior inferior temporal sulcus/middle temporal gyrus^9^, the fusiform face area (FFA) located ventrally in the fusiform gyrus, the parahippocampal place area^18^ located in the dorsal part of the gyrus parahippocampalis in the middle temporal gyrus and the visual word form area (VWFA) in the left fusiform gyrus^19^. All these locations confirm the importance of the temporal lobe in object categorization-function.

Furthermore, Epstein et al. were able to demonstrate the responsiveness of the PPA during an fMRI-session selectively to viewed scenes including single objects, but not to faces^20^.

Grill-Spector et al. confirmed the relevance of the temporal lobe in terms of visual categorization, especially the importance of the ventral part of the temporal lobe (occipitotemporal sulcus (OTS), posterior transverse collateral sulcus (ptCoS), parahippocampal gyrus (PHG) and the anterior tip of the mid-fusiform sulcus (MFS))^21^. In this area, Grill-Spector et al. point towards the localization of the high-level visual regions, which “do not process local, low-level features of visual stimuli, such as contrast or orientation, but instead process global shape and are involved in visual perception and recognition”.

The highest error rates of the current study occurred foremost in the left MFG, but also in right hemispheres´ MFG. These brain areas are also discussed in the context of attentional networks. Especially the right MFG has been proposed to be a part of the dorsal and ventral attention network in terms of reacting to an exogenous stimulus^22^. Vossel et al. pointed out that neither of the two attentional networks controls attentional processes in isolation^23^. It is rather a flexible interaction governed by the IFG and the MFG with a dynamic control of attention and interaction including the frontal, parietal, temporal and occipital lobe.

Regarding the categorization of living objects, we detected the highest error rates in bilateral parietal lobes as well as in the left temporal lobe. Haxby et al. argued, that the ventral temporal cortex plays an important role in the categorization and recognition of faces as well as objects^24^. In terms of the categorization of non-living objects we could not find significant higher ER in the temporal lobe.

In terms of categorization-function of non-living objects, the highest ER was located in bilateral frontal and parietal lobes. Grill-Spector et al. named object-selective foci involved in the recognition of objects including the lateral occipital complex (LOC) which consists of two subdivisions. First, the lateral occipital subdivision extending into the posterior inferotemporal sulcus and, second, the anterior–ventral subdivision in the posterior to midfusiform gyrus extending into the occipitotemporal sulcus^21^. Konen et al. also underlined the importance of the fusiform gyrus and therefore the temporal lobe in object-categorization by presenting a case report about a patient following an injury of the right fusiform gyrus^25^.

These findings do not confirm our results in the current study concerning the categorization of non-living objects. Finally, we point out that overall the highest ER for the wrongly categorization of living and non-living objects were just 3% error rates for all stimulations and 10% error rates for all subjects.

## Material and methods

### Ethics

Prior to every nrTMS mapping a written informed consent was signed by each participant. The local ethics committee of our university approved all aspects of the current study (Ethics Committee Registration Number 5811/13; Ethics Commission of the Technical University Munich, Ismaninger Str. 22, 81675 Munich, Germany) in accordance with the Declaration of Helsinki.

### Study subjects

Twenty healthy volunteers, who suffered from no cerebral or other pathology were enrolled. They were purely right-handed (according to the Edinburgh handedness inventory). The median age was 25.0 ± 1.7 years (range 22.0–29.5 years, Table 2). Eleven subjects were female, nine were male. Exclusion criteria according to Rossi et al.^26^ were aberrant medical history, any pathological findings on the cranial magnetic resonance imaging (MRI), medication, cardiac pacemaker, deep brain stimulation treatment in the past, developmental language deficits, cochlear implant, previous seizure or any further neurological impairment.

**Table 2:** Subjects characteristics like age, gender, correct answers during the baseline-performance, rMT and pain on the VAS.

Subject no.	Age (years)	Gender	rMT (% output)		Correct baseline pictures		Pain (VAS) convexity		Pain (VAS) temporal	
			Left	Right	Left	Right	Left	Right	Left	Right
1	23	F	28	25	25	45	2	2	5	6
2	25	M	32	39	48	57	2	3	6	6
3	29	M	37	29	55	61	2	1	6	5
4	25	M	29	25	50	56	1	1	4	7
5	23	F	27	32	46	54	0	2	4	5
6	25	M	29	28	57	62	1	1	2	2
7	24	F	35	40	34	35	2	2	4	4
8	21	M	35	31	31	45	0	1	5	3
9	26	M	37	39	38	40	5	7	6	8
10	23	F	42	33	22	26	4	1	7	5
11	24	F	38	41	33	26	4	5	7	6
12	23	F	27	27	30	27	0	2	1	6
13	23	F	40	33	46	34	2	2	3	3
14	26	M	40	33	38	34	5	4	6	7
15	26	F	39	35	29	27	1	1	3	3
16	24	F	30	29	43	41	5	5	5	7
17	24	M	30	29	20	19	4	4	7	6
18	23	F	37	32	42	33	1	2	4	3
19	27	M	35	29	58	51	2	1	3	2
20	27	F	41	32	58	54	6	4	8	5
Median	25	–	35	32	40	40.5	2	2	4.5	5
95% CI	24–25	–	32–37	30–34	35–46	35–48	1.7–3.3	1.7–3.4	4.0–5.7	4.1–5.8
P	–	–	0.997		0.694		0.975		0.992	

*f* female, *m* male, *rMT* resting motor threshold (in % stimulator output), *VAS* visual analogue scale.

Inclusion criteria were defined as age above 18 years, right-handedness, German as mother tongue and written informed consent.

### Study design

The current study was designed prospectively. Each volunteer underwent nrTMS categorization-mapping of both hemispheres in a randomized fashion. The hemispheres were examined randomly with 13–16 days delay between both mappings. The first author, who underwent nrTMS training and manufacturer certification prior to the trial to preclude learning curve effects, conducted every mapping session.

### MRI acquisition

An 8-channel phased array head coil (Achieva 3 T, Philips Medical Systems, The Netherlands B.V.) combined with a 3 Tesla MR imaging was performed prior to the first nrTMS mapping to every participant. For anatomical co-registration, the scanning protocol comprised of a three-dimensional (3D) gradient echo sequence (TR/TE 9/4 ms, 1 mm^2^ isovoxel covering the whole head, 6 min 58 s acquisition time) without intravenous contrast administration. Afterwards, using the DICOM standard, the 3D dataset was transferred to the nrTMS system.

### nrTMS mapping

#### Experimental setup

Every categorization-mapping session was performed using the Nexstim eXima NBS system version 4.3 with a NexSpeech module (Nexstim Plc, Helsinki, Finland), consisting of a biphasic figure-of-eight TMS coil in a magnetic stimulator with a radius of 50 mm as previously reported^14,27–29^. This magnetic stimulator was connected to an infrared tracking system (Polaris Spectra, Waterloo, Ontario, Canada). Each participant underwent 2 nrTMS categorization-mappings. The resting motor threshold (rMT) was defined using a motor mapping of the contralateral cortical representation of the hand area (Musculus abductor pollicis brevis, Musculus abductor digiti minimi) as described in previous studies^30,31^. In order to visually display the analog cortical area receiving nTMS pulses, a 3D T1-weighted MRI of each participant was used as an anatomically reference by a stereotactic infrared camera to track the coil position^29,32,33^. After this initial setup, the nrTMS categorization-mapping was performed using 100% rMT. The magnetic pulses were set at a frequency of 5 Hz and 10 pulses.

#### Stimulated cortical spots

**Figure 5:**
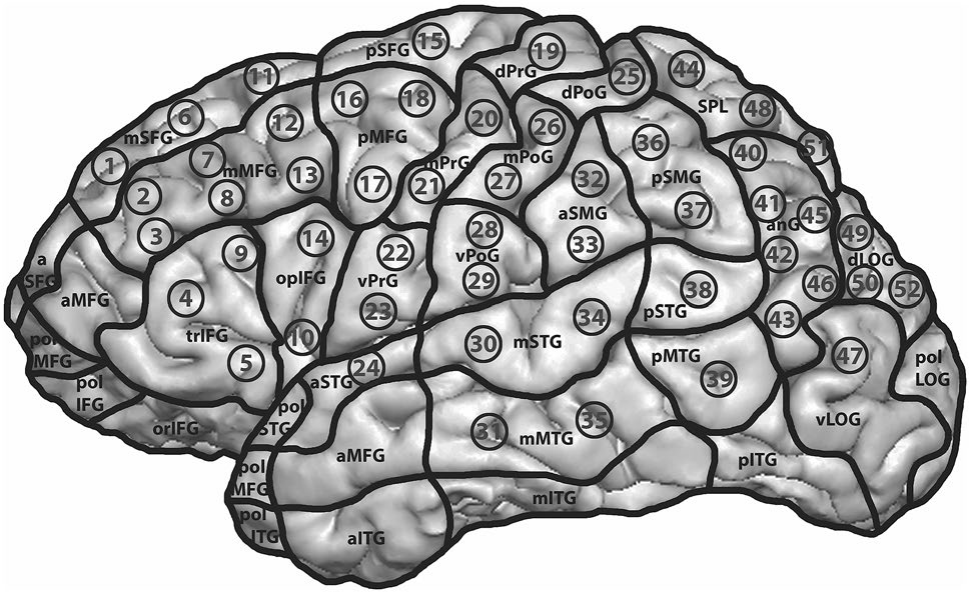
Overview of the 52 previously determined cortical spots stimulated during the categorization-mapping procedure.

While the subject was performing the categorization-mapping tasks, 52 anatomical previously determined and identified cortical spots, spread over the hemisphere, were stimulated via nrTMS (Fig. 5). The defined localizations and names of the gyri were based on the cortical parcellation system (CPS) published by Corina et al.^34^ (Table 3). Before each mapping procedure these cortical spots were added and visually marked to the MRI by the first examiner (Fig. 5). Each of the 52 spots was stimulated for three times with an electric field strength at a cortical level ranging between 55 and 80 V/m. After the third stimulation of a cortical spot, the coil was relocated to the next spot according to their numerical order. During the stimulation the coil was positioned tangentially to the subject’s skull in strict anterior–posterior field orientation^10,35^. Some cortical spots were excluded from the mapping procedure because of the disagreeableness their stimulation could cause or due to their accessibility for the nrTMS stimulation coil^14,15^. The spots, which were excluded from nrTMS were located in the anterior middle temporal gyrus (aMTG), the inferior temporal gyrus (ITG), the polar superior temporal gyrus (polSTG), the inferior frontal gyrus (IFG), the middle frontal gyrus (MFG) and the orbital part of the inferior frontal gyrus (orIFG).

**Table 3:** Cortical parcellation system.

Abbreviation	Anatomy
aITG	Anterior inferior temporal gyrus
aMFG	Anterior middle frontal gyrus
aMTG	Anterior middle temporal gyrus
anG	Angular gyrus
aSFG	Anterior superior frontal gyrus
aSMG	Anterior supramarginal gyrus
aSTG	Anterior superior temporal gyrus
dLOG	Dorsal lateral occipital gyrus
dPoG	Dorsal post-central gyrus
dPrG	Dorsal pre-central gyrus
mITG	Middle inferior temporal gyrus
mMFG	Middle middle frontal gyrus
mMTG	Middle middle temporal gyrus
mPoG	Middle post-central gyrus
mPrG	Middle pre-central gyrus
mSFG	Middle superior frontal gyrus
mSTG	Middle superior temporal gyrus
opIFG	Opercular inferior frontal gyrus
orIFG	Orbital part of the inferior frontal gyrus
pITG	Posterior inferior temporal gyrus
pMFG	Posterior middle frontal gyrus
pMTG	Posterior middle temporal gyrus
polFG	Polar frontal gyri
polTG	Polar temporal gyri
polLOG	Polar lateral occipital gyrus
pSFG	Posterior superior frontal gyrus
pSMG	Posterior supramarginal gyrus
pSTG	Posterior superior temporal gyrus
SPL	Superior parietal lobe
trIFG	Triangular inferior frontal gyrus
vLOG	Ventral lateral occipital gyrus
vPoG	Ventral post-central gyrus
vPrG	Ventral pre-central gyrus

Anatomical names and abbreviations of the cortical areas according to Corina et al.^34^.

#### Categorization tasks

The subjects were instructed to solve simple categorization tasks. They consisted of 80 pictures in total (40 living and 40 non-living images). The living images included pictures of 40 different animals in color in front of a white screen. The non-living pictures consisted of 40 different objects in color of everyday life, such as tools, a clock, a suitcase, a dress, dice etc. These images were presented at random on a 15-inch screen 60 cm in front of the volunteer.

#### Categorization mapping procedure

Ahead of every categorization-mapping procedure, the subjects underwent a baseline measurement consisting of the previously mentioned 80 pictures of living and non-living objects displayed on a screen in front of them. The baseline was performed without any nrTMS or sham stimulation. The answers had to be given as fast as possible, accurately, and without any incorrect pronunciation or stuttering. All falsely categorized, wrongly pronounced or misnamed pictures were counted and excluded from the stimulus sequence (Table 2). Each living or non-living object was displayed for 700 ms with a fixed inter-picture interval (IPI) of 3 s and 0 ms picture-to-trigger interval. Exactly the same modalities were used for the baseline performance as well as for the categorization-mapping procedure. During the categorization-mapping procedure, the volunteers had to categorize the living or non-living objects in German while nrTMS pulses were applied. The whole mapping session, including the baseline performance, was video recorded for objective post-hoc analysis. We were therefore able to compare every given answer under stimulation with the baseline afterwards. Local nrTMS-induced pain in temporal brain regions and the remaining hemisphere (convexity) was evaluated via a visual analogue scale (VAS) (Table 2).

### Video data analysis

The analyzer was blinded to the stimulated cortical spots as well as the previous results in every case. The evaluation of the recorded nrTMS categorization sessions was performed as described in earlier studies^27,36–38^. At the beginning, the baseline performance was analyzed, followed by the performance of the categorization-mapping during stimulation. All errors, such as falsely categorized tasks were compared to the baseline. Thereby we tried do differ precisely between speech-related errors or categorization-related impairment. If at least one out of three stimulated cortical spots evoked an error, the spot was considered as error positive in terms of the categorization mapping. In total, the nrTMS induced errors were categorized into:

- All generated errors during the categorization-mapping procedure
- No categorization possible (no answer during the time of stimulation). This error category needs to be added primarily to speech-related errors.

Hesitation errors (delayed answer after stimulation onset). Hesitation errors are a common error category used to detect positive cortical localizations and fibers in the white matter during a speech related mapping. Recording all made mistakes during the categorization mapping, we kept analyzing all mistakes as well, while trying to differ between the sources of the development of the different errors.

- Wrongly categorized living objects
- Wrongly categorized non-living objects.

The error rate (ER) is the quotient of the number of nrTMS-induced categorization task errors, divided by the number of categorization tasks and nrTMS stimulations in total. These ER were analyzed in two different ways:

1. ER for all errors per total number of stimulations of the whole mapping procedure; this ER shows the actual mistakes made per specified category in percentage.
2. ER concerning all subjects who generated mistakes per all stimulated subjects.

### Statistical analysis

For testing the differences between the two hemispheres in ER we used a Mann–Whitney–Wilcoxon test for multiple comparisons on ranks for independent samples for non-parametric distributions. In this case, the ER for all errors of all subjects were separated for the both types of categorization (living, non-living) and compared in the left versus the right hemisphere.

In terms of testing the various attributes, a Chi-square test was performed. In this context, all errors of all stimulated cortical spots in the entire categorization mapping in the left and the right hemisphere were compared. For comparing the ER for all errors of all subjects in one hemisphere, we used the ANOVA Kruskal–Wallis test.

The results are presented as odds ratios (OR) with 95% confidence intervals (CI) (GraphPad Prism 7, La Jolla, CA, USA).

## Limitations

One important fact about the detection of cortical function is the limitation of nrTMS due to its limitation to achieve all cortical areas. For example, some important areas for cortical categorization function are located in not well reachable areas for the stimulation coil, such as the inferior temporal gyrus. Nevertheless, with the cortical map of observed categorization positive spots, we are able to create a fiber tracking and therefore to display the important tracks in the white matter^39,40^.

Direct cortical stimulation is still the gold standard in intraoperative monitoring. Yet, there is no gold standard in examining the categorization function of the brain. It could be a possibility for future studies, to examine cortical categorization-function intraoperatively with direct cortical stimulation in awake craniotomy.

Another frequently discussed limitation of nrTMS and the mapping of neuropsychological function or language function is the possible mapping of adjacent cortical areas, because of their functional connectivity. Moreover, during the video-analysis it can be difficult to differentiate, whether the mistake was generated by categorization impairment or language impairment, such as in the left hemisphere’s IFG. A possibility to differentiate between language-related errors or categorization-related errors might be a language control task in further studies in which subjects would perform a categorization mapping, as well as a language mapping. Afterwards, the examined cortical spots could be compared. In the current study, the highest ER for all errors was generated in the left hemisphere’s mMFG and therefore not in typical language related areas.

This study was the first step to examine the feasibility of nrTMS to map cortical categorization function. It is debatable, whether the resection of the left mMFG leads to impairment of categorization function or to clinical deficit.

Another important fact is the number of examined subjects. An objective for upcoming studies is to increase the number of participants to reach a higher statistical significance and to implement a test–retest analysis.

Last, in terms of hesitation errors, there was no exact reaction time measurement, as they were only compared to the baseline testing after the mapping by the examiner. In the context of this specific error category, there is still need of improvement due to the analyzing techniques.

## Conclusion

nrTMS seems feasible for the detection of cortical categorization function. Moreover, the observed bihemispheric representation, as well as the higher error incidence for the categorization of non-living objects, is well in accordance with current literature. The major limitation of this technique is that some important areas for cortical categorization function are located in not well reachable areas for the stimulation coil, such as the inferior temporal gyrus.

## Data Availability

The local ethics committee of our university approved all aspects of the current study (Ethics Committee Registration Number 5811/13) in accordance with the Declaration of Helsinki. All authors contributed to the study conception and design. Material preparation, data collection and analysis were performed by Stefanie Maurer and Sandro Krieg. The first draft of the manuscript was written by Stefanie Maurer and all authors commented on previous versions of the manuscript. All authors read and approved the final manuscript.

## Abbreviations

3D: Three-dimensional
anG: Angular gyrus
aMTG: Anterior middle temporal gyrus
CPS: Cortical parcellation system
EBA: Extrastriate body area
ER: Error rate
FFA: Fusiform face area
IFG: Inferior frontal gyrus
ITG: Inferior temporal gyrus
MFG: Middle frontal gyrus
MFS: Anterior tip of the mid-fusiform sulcus
mMFG: Middle middle frontal gyrus
mMTG: Middle middle temporal gyrus
mPoG: Middle post-central gyrus
mPrG: Middle pre-central gyrus
MRI: Magnet resonance imaging
mSTG: Middle superior temporal gyrus
nrTMS: Navigated repetitive transcranial magnetic stimulation
nTMS: Navigated transcranial magnetic stimulation
opIFG: Opercular inferior frontal gyrus
orIFG: Orbital part of the inferior frontal gyrus
OTS: Occipitotemporal sulcus
PHG: Parahippocampal gyrus
pMFG: Posterior middle frontal gyrus
polSTG: Polar superior temporal gyrus
PPA: Parahippocampal place area
SMA: Supplementary motor area
pSMG: Posterior supra marginal gyrus
ptCoS: Posterior transverse collateral sulcus
rMT: Resting motor threshold
SP: Stimulation point
SPL: Superior parietal lobe
trIFG: Triangular inferior frontal gyrus
mMTG: Middle middle temporal gyrus
vPoG: Ventral post-central gyrus
VWFA: Visual word form area
